# Making Morphology Meaningful: Tracking Dynamic Catalyst Evolution under Working Conditions

**DOI:** 10.1021/acsami.6c04016

**Published:** 2026-04-20

**Authors:** Shih-Wei Cheng, Feng-Ze Tian, Guan-Bo Wang, Hao Ming Chen

**Affiliations:** † Department of Chemistry, 33561 National Taiwan University, Taipei 106, Taiwan; ‡ National Synchrotron Radiation Research Center, Hsinchu 300, Taiwan; § Center for Emerging Materials and Advanced Devices, National Taiwan University, Taipei 10617, Taiwan

**Keywords:** morphology control, surface reconstruction, in situ/operando characterization, structure−activity relationship, catalysis

## Abstract

Morphology control has long been a widely adopted strategy in catalyst design, based on the assumption that geometric and electronic structures are inseparable in determining catalytic behavior. This perspective revisits that assumption by highlighting how catalyst structures dynamically evolve under realistic reaction conditions. Traditional structural descriptors, including local structural metrics such as coordination number and morphology-related features such as facet exposure, are commonly derived from the as-synthesized state and may not reflect the actual active configurations during reactions. Evidence from photocatalytic, thermocatalytic, and electrocatalytic systems reveals that catalysts undergo significant structural and electronic transformations in response to illumination, thermal input, and applied potential. These findings challenge the conventional static structure–activity paradigm and call for a revised framework that integrates morphology control with in situ characterization techniques. By tracking structural, electronic, and compositional changes in real time, morphology can be redefined not as a fixed geometric label but as an evolving platform that connects initial design to dynamic catalytic function.

## Graphical abstract

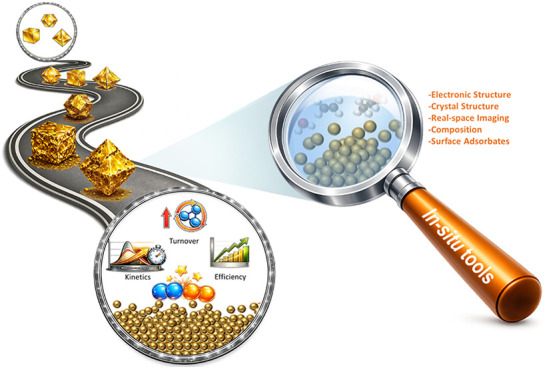

The global revenue of the chemical industry exceeds US$6.2 trillion annually,^1^ and an estimated 85% of these processes depend on catalysis,^2^ in which catalysis refers to the use of catalysts to alter the rate and/or pathway of a chemical reaction without being consumed in the process. Its essential function lies in providing an alternative reaction route with a lower activation energy, thereby enabling transformations to proceed at milder temperatures and pressures. Beyond mere acceleration, a catalyst acts as a critical “gatekeeper”, determining the thermodynamic and kinetic feasibility of specific pathways under practical operating conditions.^3^ The transformative power of catalysts is evident from history to the present day. Classical examples include the iron-catalyzed Haber–Bosch process, which revolutionized global food production, and catalytic cracking, which reshaped petroleum utilization.^3^ This transformative impact is also evident in modern energy and environmental technologies, where platinum group metals have made fuel cells viable and automotive catalytic converters have dramatically reduced air pollution. Today, catalysts continue to play pivotal roles in addressing urgent energy and environmental challenges, such as driving CO_2_ electroreduction,^4^ enabling green hydrogen generation,^5^ and facilitating sustainable ammonia synthesis.^6^ These examples highlight that catalysts do more than merely accelerate chemistry; they govern the trajectory of global energy transitions and the feasibility of sustainable technologies.

Since catalysis underpins such critical transformations, the demands placed upon catalysts are becoming more stringent. Conventional discovery strategies, long guided by empirical trial-and-error, offer limited predictive power and cannot meet the growing demand for efficient and sustainable catalysts.^7,8^ This limitation has driven a decisive shift in catalysis research away from accidental discovery toward rational and principle-based design. Achieving such rational design, however, requires a deep mechanistic understanding of how catalysts facilitate chemical transformations. Catalytic reactions fundamentally rely on the ability of catalysts to mediate charge distribution between reactants and active sites, thereby lowering activation barriers and steering reaction pathways.^9,10^ The catalytic activity is inherently dependent on the alignment between the electronic states of catalysts and the adsorbate orbitals, a relationship that governs charge redistribution and bond activation. Specifically, the d-band theory provides a quantitative framework linking the electronic structure of surfaces to their reactivity.^11^ In dynamically evolving catalytic systems, this relationship may also vary over time as local coordination environments continuously change. Such changes can lead to corresponding shifts in electronic structure and d-band characteristics.

At the atomic scale, catalysis occurs at discrete active sites where geometric coordination and local coupling between the adsorbate frontier orbitals and the transition-metal *d*-states define adsorption energies and reaction barriers. This relationship underlies the conceptual understanding of how variations in electronic coupling translate into systematic trends in catalytic activity.^12^ Incorporating site-specific geometric effects into these relations further reveals that changes in local coordination can substantially alter adsorption strength and reaction energetics.^13^ Consequently, the interaction between reactive centers and reactants is central to catalytic function and provides the foundation for understanding how catalysts can be rationally tuned. Rational catalyst design requires understanding the factors that govern the interaction between active sites and adsorbates.^14^ Intrinsically, the elemental composition and electronic structure dictate how the metal *d*-states couple with adsorbate orbitals, thereby tuning adsorption strength and reactivity.^11,15^ In this case, the specific configuration of surface atoms, including coordination number, facet orientation, and defect density, defines the local electronic environment and charge distribution. For instance, exposing high-index or concave facets may introduce low-coordination atoms that facilitate stronger orbital overlap and modulate interfacial charge distribution.^16,17^ Beyond intrinsic and geometric factors, the interaction between the active phase and its support can profoundly reshape electronic properties. Intrinsic metal–support interactions can redistribute charge across the interface, modulating both activity and stability through electronic coupling and structural reconstruction.^18,19^

These factors define the multidimensional landscape that governs reactant–active site interactions and, consequently, catalytic performance. In this context, these factors encompass both geometric effects, which define the spatial arrangement of active sites, and electronic effects, which govern adsorption energetics through modifications of the local electronic structure. Among these multidimensional factors, the atomic configuration of catalysts plays a decisive role in determining the nature of adsorbate interplay with active sites. Geometric descriptors, such as the coordination number, capture the intrinsic connection between surface structure and reactivity; atoms with lower coordination exhibit unsaturated bonds and higher chemical reactivity because their electronic density enables stronger coupling with adsorbates.^8,13,20^ Consequently, facets, edges, and defects constitute distinct classes of active sites whose distribution defines overall catalytic behavior. Understanding how these geometric factors control charge distribution provides the conceptual foundation for rational catalyst design.^8^ Building upon this conceptual framework, understanding and controlling the structural factors that govern charge distribution offer not only a theoretical basis for catalysis but also a practical route toward the rational design of high-performance catalysts. Among these various structural parameters, morphology control represents a particularly powerful dimension of catalyst design because it bridges atomic configuration with the macroscopic manifestation of catalytic activity. By translating atomic-level insights into controllable structural motifs, morphological design offers a tangible means to realize the principles outlined above.

## Morphology Control as a Dimension of Catalyst Design

In this perspective, we distinguish between “structure” and “morphology” as related but distinct concepts. Structure refers to the local atomic configuration of a catalyst, including coordination environment, bonding arrangement, and electronic state, whereas morphology describes the higher-level geometric manifestation of how these atomic features are collectively expressed, such as overall shape, facet exposure, and mesoscale form. At the level of active sites, geometric and electronic structures are inseparable in defining catalytic behavior, and the structural sensitivity of catalytic activity originates from variations in local coordination and bonding environments.^21^ Geometric descriptors, such as the coordination number or its generalized formulations, provide quantitative links between atomic arrangement and catalytic behaviors by correlating surface atom configurations with adsorption energetics and charge distribution.^20^ As illustrated in Figure 1a, the distinct atomic packing of low-index facets, such as the square geometry of (100), the rectangular rows of (110), and the close-packed triangular lattice of (111), represents unique coordination environments that determine surface energy and growth preference. The relative exposure of these facets defines the morphology of nanoparticles (NPs), generating distinct ensembles of active sites that underpin morphology-dependent reactivity.^22^ For instance, Cu surfaces with various step densities and terrace arrangements exhibit markedly different product selectivity during CO_2_ electroreduction, directly reflecting how atomic configuration governs morphology-specific catalytic outcomes.^23^ Furthermore, defects, often manifested as undercoordinated atomic sites and intentionally introduced through defect engineering, act as localized structural motifs that tailor charge distribution and modulate interactions between reactive centers and reactants.^24^ Viewed in this way, morphology can be understood as the macroscopic manifestation of atomic configuration, offering a clear conceptual basis for relating geometric anisotropy to catalytic performance.

**Figure 1:**
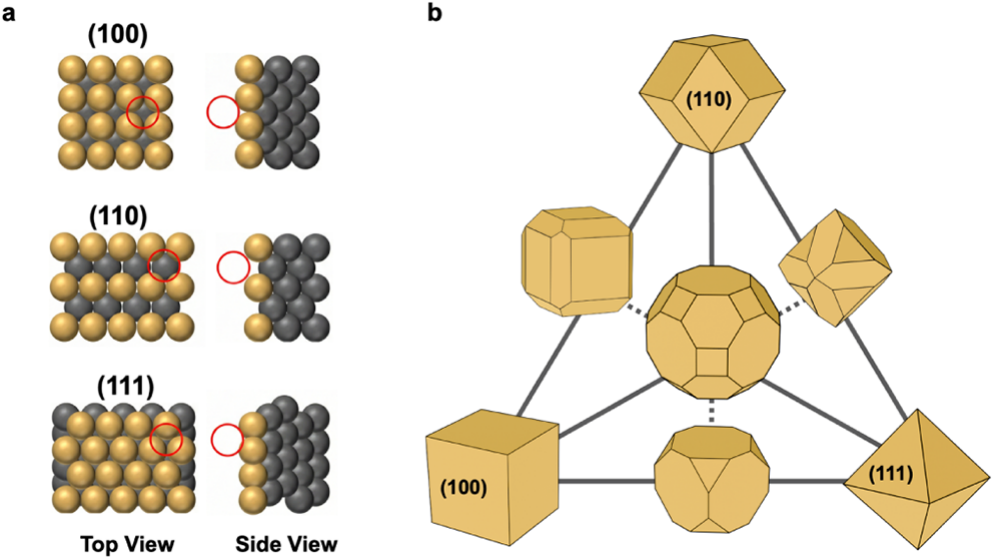
Relationship between atomic configuration and catalytic morphology. (a) Distinct atomic arrangements of low-index surfaces of fcc lattices define local coordination environments. (b) Wulff construction illustrates how these facets combine to yield characteristic equilibrium morphologies.

Morphology can be viewed as the macroscopic manifestation of how atoms organize at the nanoscale to minimize surface energy. In metallic and oxide catalysts, the overall shape of NPs arises from the relative stability of low-index crystallographic planes, each exhibiting distinct coordination and surface energy characteristics.^25^ As illustrated in Figure 1b, the Wulff construction provides a theoretical framework describing how these facets combine to produce well-defined morphologies such as cubes, octahedra, and rhombic dodecahedra, whose exposed surfaces define the distribution of catalytically relevant sites.^26^ It should be noted, however, that such equilibrium-based descriptions, including the Wulff construction, are strictly valid only under ideal equilibrium conditions and may not describe kinetically trapped morphologies commonly obtained in nanoparticle synthesis. In practice, the label of a given morphology reflects a statistical ensemble of facets and defect motifs rather than a single ideal surface, so macroscopic shape should be interpreted as the collective exposure of diverse coordination environments.^27^ This facet and defect exposure dictates how adsorbates approach and bind to the surface, how reaction intermediates and transition states are stabilized, and how charge distribution proceeds at active sites, thereby setting trends in catalytic activity and selectivity across reactions.^25,28^ Viewed in this way, morphology defined under thermodynamic equilibrium conditions provides a clear geometric basis for rational catalyst design. By linking atomic-scale coordination environments with macroscopic geometric anisotropy, morphology serves as a central bridge between catalyst structure and function.^28^ However, the current scientific debate is not about dismissing the importance of morphology control, but rather about reevaluating when morphology serves as a valid structural descriptor. The effectiveness of this descriptor depends on several key factors, including the reaction driving force, material class, the strength of support interactions, and the thermochemical or electrochemical window of operation. From a mechanistic perspective, the interpretive value of morphology has clear applicability boundaries. In stable regimes with low driving forces, where surface atoms do not undergo significant reconstruction or atomic rearrangement, the initial morphology directly determines active site distribution and retains predictive power. However, under realistic operating conditions, morphology is no longer a static indicator but instead becomes a starting state that defines how the catalyst evolves over time. Therefore, morphology control should not be viewed as a fixed geometric label, but rather as a dynamic parameter shaped by the reaction environment. Ignoring this conditional validity is a primary reason why static models often fail to match experimentally observed behavior under working conditions.

## Atomic Configuration May Change across Catalytic Platforms

In general, a common workflow in catalysis research involves synthesizing nanocrystals with precisely controlled morphologies and correlating their catalytic performance with theoretical calculations based on the as-synthesized structure. It should be noted that this strategic approach relies on a critical assumption that the initial morphology of the catalyst remains intact under realistic reaction conditions, allowing static theoretical models to faithfully rationalize experimental observations. Nevertheless, the validity of this assumption under operating catalytic environments has increasingly been debated. In practice, the morphological identity of catalysts in their “working state” may differ from those in their “as-synthesized” state. Therefore, theoretical predictions based solely on the pristine structure may not accurately reflect the phenomena governing catalytic behavior under reaction conditions. Photocatalysis provides a representative demonstration of this limitation. In photocatalytic systems, the absorption of photons generates electron–hole pairs that migrate to the catalyst surface and drive redox transformations. In contrast to purely photocatalytic processes, photoelectrochemical systems operate under simultaneous illumination and applied bias, introducing a strongly nonequilibrium reaction environment that can further destabilize the initial morphology and accelerate structural reorganization at both surface and interface. As a result, morphological evolution becomes even more pronounced under photoelectrochemical conditions. At a conceptual level, both photocatalytic and photoelectrochemical systems can be viewed as photon-driven processes, as light absorption initiates charge carrier generation.

In a typical photocatalytic study, Cu_2_O nanocrystals with well-defined cubic, octahedral, and rhombic dodecahedral morphologies were employed as model surfaces for examining facet-dependent photocatalytic degradation of methyl orange (Figure 2a). In this framing, morphology appears to be a static determinant of reactivity. Yet microscopic characterization before and after reaction reveals that these ideal morphologies are not preserved, in which surface roughening and facet rounding occur through the reaction across all shapes, indicating that illumination induces continuous reconstruction of surface structures. Even more pronounced structural evolution is evident in photoelectrochemical systems, where illumination is combined with an applied potential, introducing additional driving forces for atomic rearrangement at both surface and interfacial regions (Figure 2b–f). During photon-assisted CO_2_ electroreduction, Cu nanocubes (NCs) exhibit substrate-dependent variations in ethylene formation (Figure 2c). Cu supported on black silicon (BSi) undergoes atomic interdiffusion across the Cu–Si junction, forming an interfacial Cu_3_Si silicide layer accompanied by expansion of the Cu lattice and partial oxidation of surface atoms (Figure 2d). High-resolution transmission electron microscopy (HR-TEM) images further reveal redeposition of mobile Cu atoms onto exposed BSi regions (Figure 2e), confirming that Cu species dissolve, migrate, and renucleate within the electrolyte during reaction. Operando Fourier-transformed EXAFS measurements demonstrate potential-dependent variations in Cu coordination features, with Cu NCs on BSi showing larger Cu–Cu peak shifts than those on glassy carbon, indicative of a more pronounced dynamic structural response at the semiconductor interface (Figure 2f). These results suggest that photons and electrons can simultaneously drive both interfacial alloying and surface reorganization under light-assisted catalytic conditions. Consequently, illumination induces dynamic reorganization across photocatalytic and photoelectrochemical systems, such that the atomic configurations under reaction conditions deviate from the as-synthesized state.

**Figure 2:**
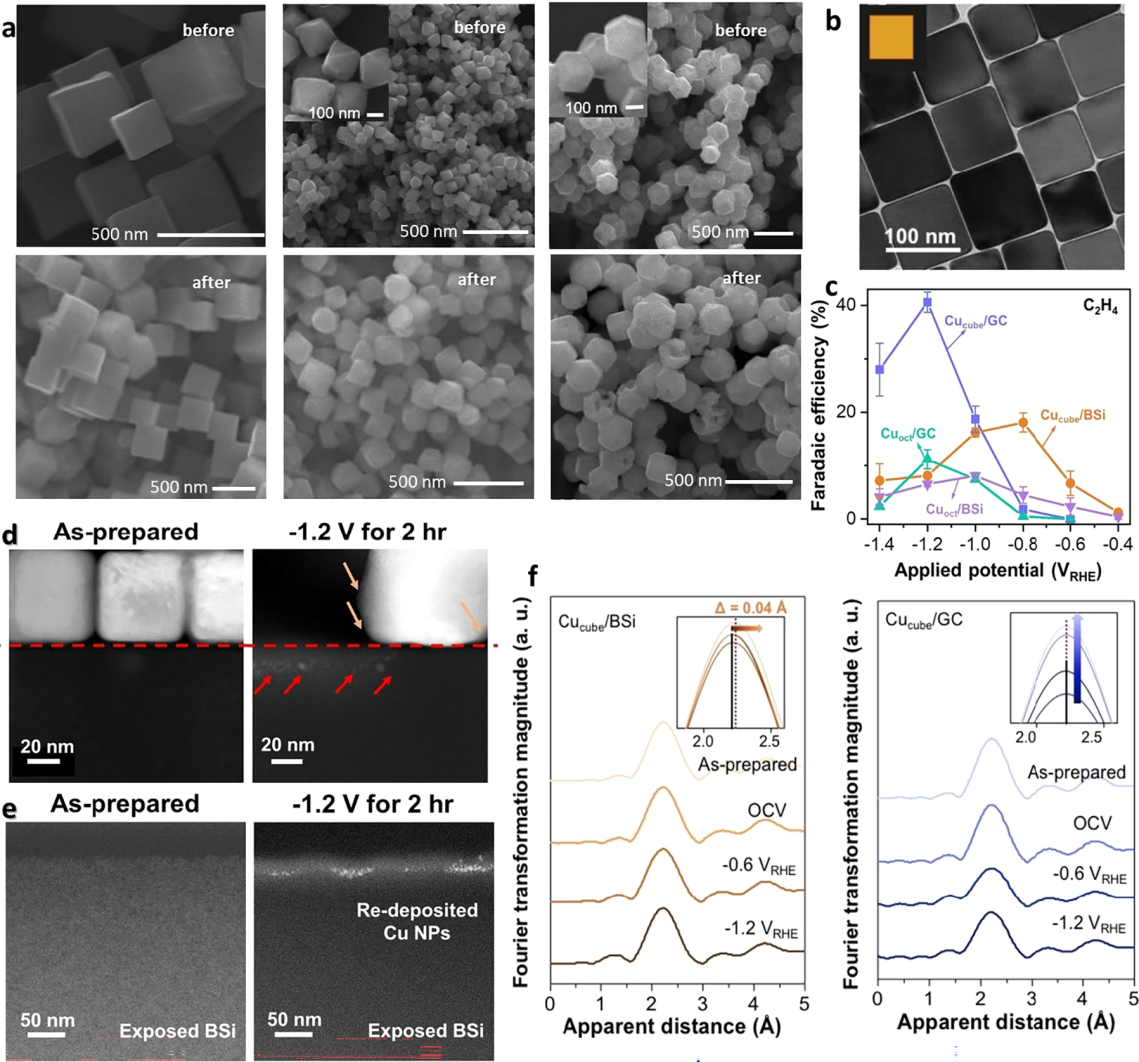
Photon-driven configuration evolution in photocatalytic and photoelectrocatalytic systems. (a) SEM images showing facet-dependent morphological evolution of Cu_2_O nanocrystals before and after photocatalytic reaction (Adapted with permission from ref. 29. Copyright 2017 the Royal Society of Chemistry). (b–f) Light-induced dynamic restructuring of Cu NCs supported on black silicon (BSi) during CO_2_ reduction. (b) TEM image of as-synthesized Cu NCs. (c) Faradaic efficiencies of C_2_H_4_ formation on Cu NCs supported on different substrates. (d–e) Cross-sectional images displaying interfacial Cu-silicon interdiffusion and redeposited Cu particles on exposed BSi regions after reaction. (f) Operando Fourier-transformed EXAFS spectra displaying potential-dependent Cu–Cu coordination shifts on black silicon compared to those on glassy carbon (Adapted with permission from ref. 30. Copyright 2024 Wiley-VCH GmbH). Collectively, these results show that photon-driven catalytic systems can induce both surface roughening and interfacial alloying, making the operative state distinct from the prereaction morphology.

On the other hand, thermocatalysis subjects materials to elevated temperatures and reactive gas atmospheres that continuously perturb surface and subsurface atomic arrangements. Under such conditions, even geometrically well-defined metal nanocrystals undergo spontaneous and periodic restructuring as reaction intermediates modulate local coordination environments. As a well-established and particularly well-resolved model system in thermocatalysis, shape-controlled Pd nanocrystals offer a clear demonstration of dynamic coupling between morphology and reactivity during CO oxidation (Figure 3a–d). NCs enclosed by {100} facets retain their cubic geometry up to 460 °C and exhibit stable CO and CO_2_ signals without oscillatory behavior (Figure 3a–b). In contrast, the {111}-dominated octahedra display rhythmic fluctuations in CO and CO_2_ partial pressures as temperature cycles between 300 and 380 °C (Figure 3c–d), accompanied by reversible rounding and flattening of corner facets. These periodic changes originate from CO-coverage-mediated periodic transitions between high-index active surfaces and low-index inactive states, linking surface reconstruction directly to reaction-rate modulation.

**Figure 3:**
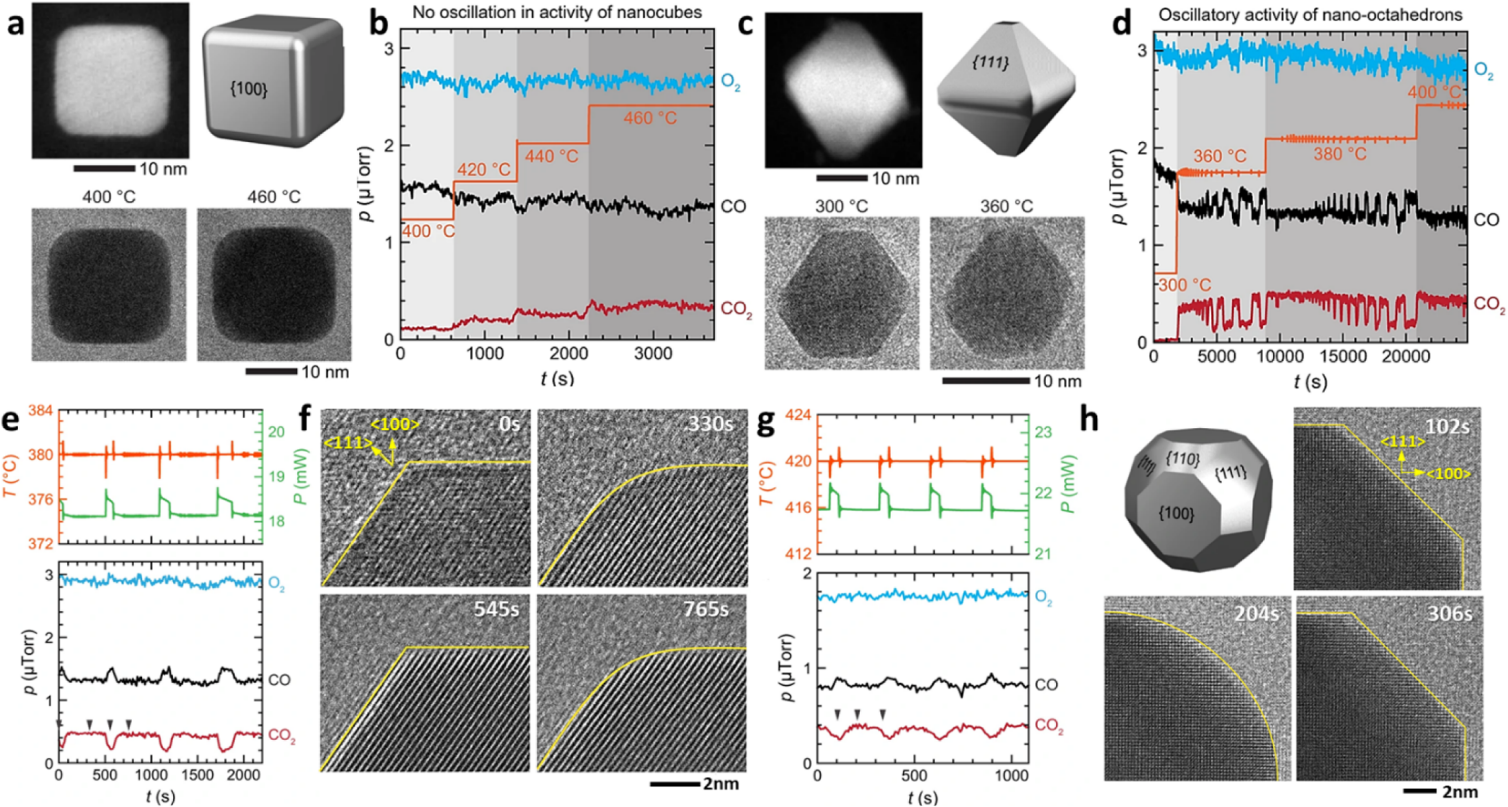
Temperature-driven periodic structural changes in thermocatalysis. (a–d) Facet-dependent dynamics of Pd NCs and octahedra during CO oxidation. Cubes remain structurally stable, whereas octahedra display oscillatory CO and CO_2_ signals coupled to reversible facet reconstruction. (e–h) In situ TEM and gas-phase analysis reveal synchronized atomic rearrangements and reaction-rate oscillations, establishing morphology–activity coupling in thermocatalysis (Adapted from ref. 31. Available under a CC-BY 4.0 license. Copyright 2022 The Author(s)). This figure illustrates a reversible oscillatory evolution mode in which morphology and activity covary dynamically.

At atomic resolution, in situ heating experiments reveal that each oscillation cycle involves rapid step-edge migration and facet reorganization occurring within seconds (Figure 3e–h). Temperature and gas-phase profiles tracking temperature (T), power input (P), and partial pressures of CO, CO_2_, and O_2_ show synchronized oscillations, confirming that structural dynamics are autocatalytically sustained through feedback between exothermic CO oxidation and oxygen replenishment. Although the overall polyhedral outline of Pd particles persists, their surfaces continuously “breathe”, expanding and contracting with each reaction cycle. These observations reveal that, under realistic thermocatalytic environments, thermocatalysts behave as dynamic ensembles rather than static solids: facets interchange between active and inactive states, lattice strain accumulates and relaxes, and catalytic turnover becomes synchronized with structural breathing modes. Corroborating with light-assisted systems, the atomic structures governing catalytic turnover in thermocatalysis fundamentally differ from the as-synthesized states and evolve continuously in response to reaction dynamics.

Similar morphological evolution has also been documented in electrocatalytic environments, where reaction conditions introduce persistent structural perturbations during operation. For example, time-resolved ex-situ electron microscopy of shape-controlled Cu NCs reveals a progressive loss of the initial cubic morphology during electrochemical CO_2_ reduction, driven by potential-induced nanoclustering and aggregation over extended reaction times (Figure 4a–f). In electrocatalytic systems, externally applied potentials impose rapidly changing redox and interfacial environments. Electrocatalysts are thus exposed not only to reactants/adsorbates but also to ion fluxes, local pH gradients, and stresses associated with gas evolution, all of which drive continuous atomic-scale structural reorganization. Atomic configurations in electrocatalysts are inherently dynamic, continuously evolving in response to the redox chemistry, electric-field effects, and mass-transport processes in the electrochemical environment. Consistent with this scheme, Cu-based electrocatalysts for electrochemical CO_2_ reduction experience structural rearrangements spanning multiple length scales, as evidenced by operando X-ray absorption spectroscopy (XAS), liquid-phase electron microscopy, and X-ray diffraction (XRD). Reversible oxidation–reduction cycling between Cu^0^ and Cu^+^ induces periodic surface reconstruction, forming transient active phases that correlate with product selectivity in CO_2_ reduction.^32–36^

**Figure 4:**
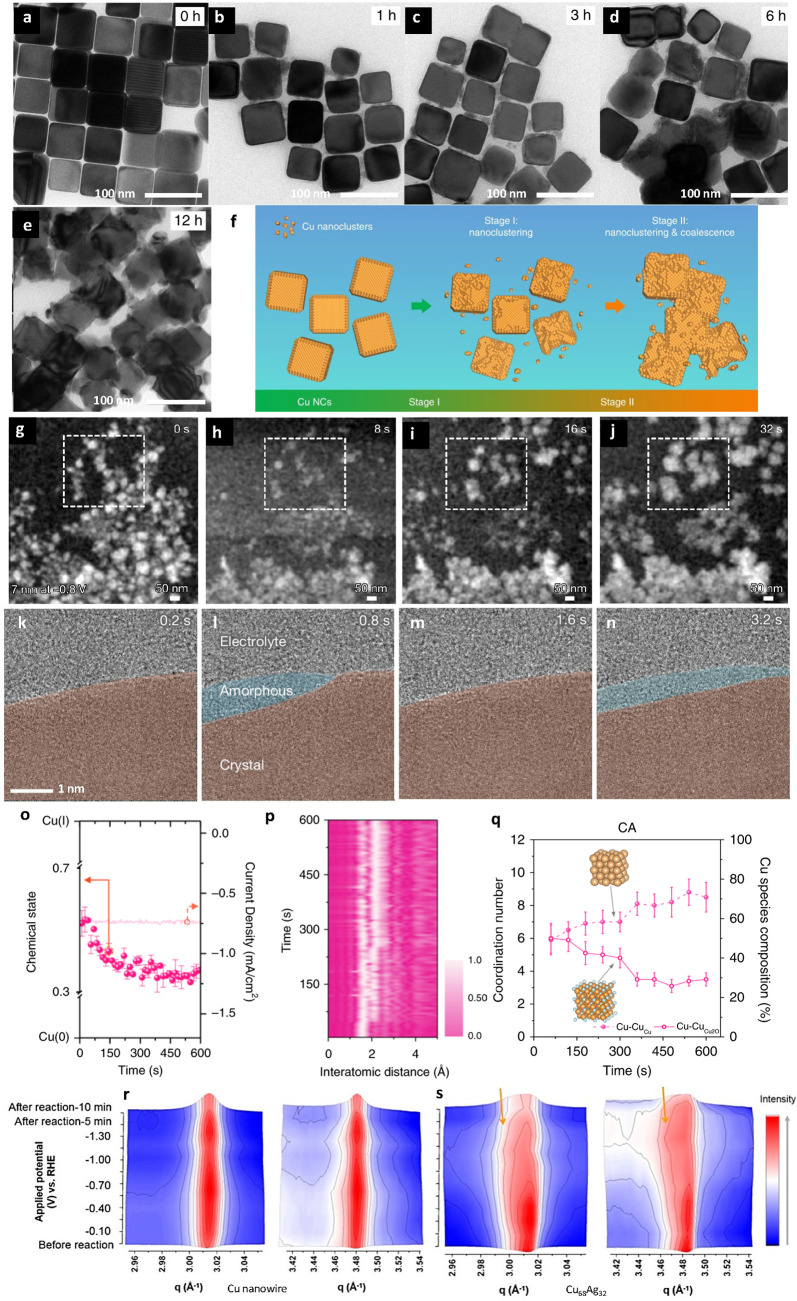
Operando and time-resolved visualization and spectroscopic tracking of dynamic structural evolution in Cu-based electrocatalysts during CO_2_ reduction. (a–e) Ex-situ TEM images of Cu NCs after different electrolysis durations under an applied potential of −1.1 V. (f) Proposed mechanism of Cu NCs under an applied potential of −1.1 V through ex-situ TEM images (Adapted from ref. 39. Available under a CC-BY 4.0 license. Copyright 2018 The Author(s)). (g–j) Liquid-cell electron microscopy images showing rapid particle migration, aggregation, and coalescence of Cu NPs under an applied potential of −0.8 V, with dashed boxes highlighting regions undergoing pronounced structural rearrangement (Adapted with permission from ref. 40. Copyright 2023 Springer Nature). (k–n) Liquid-phase transmission electron microscopy images capturing the emergence and evolution of a transient amorphous interfacial layer at the electrified Cu surface (Adapted with permission from ref. 41. Copyright 2024 Springer Nature). (o–p) Cu K-edge quick XAS measurements and corresponding time-resolved EXAFS analyses were performed during chronoamperometric operation. (q) Coordination numbers and Cu species fractions obtained from EXAFS fitting of the time-resolved spectra (Adapted from ref. 42. Available under a CC-BY 4.0 license. Copyright 2020 The Author(s)). (r–s) Operando X-ray absorption and scattering measurements on CuAg alloys capture coordinated electronic and lattice rearrangements under applied potential, indicating that configuration evolution extends from single-atom systems to bimetallic catalysts (Reproduced from ref. 43. Copyright 2020 American Chemical Society). This figure highlights that Cu electrocatalysts reconstruct across multiple length scales, from particle morphology to local coordination and interfacial phases.

Liquid-phase TEM directly captures rapid particle migration, aggregation, and coalescence during electrolysis, indicating that the working surface is continuously reshaped on short time scales (Figure 4g–j). Beyond particle-level rearrangement, liquid-phase TEM further reveals the formation of transient amorphous interfacial layers that flow along the copper surface and reversibly interchange underlying crystalline regions, highlighting the highly dynamic nature of the electrified solid–liquid interface (Figure 4k–n). These observations establish that nanoscale restructuring and atomic-scale rearrangements are intrinsically coupled during electrolysis. Beyond visually apparent morphological rearrangements, these dynamics are accompanied by time-dependent evolution in chemical state and local coordination, as resolved by quick time-resolved XAS. The Cu chemical state evolves rapidly and approaches a steady condition during operation rather than progressing monotonically to a single static valence (Figure 4o). In parallel, time-resolved EXAFS signatures change continuously with reaction time, evidencing ongoing reconfiguration of the local bonding environment (Figure 4p). Quantitative analysis further shows the systematic evolution of multiple structural parameters during electrolysis, including coordination metrics, Cu–Cu contributions, and the relative composition of Cu species, demonstrating that the operative Cu configuration is dynamically established only under working conditions (Figure 4q). These spectroscopic signatures quantitatively complement the morphological evolution observed by electron microscopy, linking long-term shape changes to continuous reconfiguration of local coordination environments. Operando XRD further indicates that crystallographic configurations also adapt to the reaction environment, such as copper-based bimetallic systems undergo potential-induced adjustments within their lattice (Figure 4r–s). Consequently, these studies demonstrate that copper catalysts do not retain their initial configurations during reaction but instead evolve through potential-driven reorganization, a behavior that is not unique to Cu but has been observed across a wide range of electrocatalytic materials.^37,38^

Crucially, such structural evolution should not be interpreted simply as material degradation or as a contradiction to thermodynamic stability. In many electrocatalytic systems, such as the Cu-based catalysts discussed above, the bulk phase may remain thermodynamically stable within the corresponding Pourbaix diagram, yet the surface can undergo substantial reorganization under operating conditions. This apparent discrepancy arises because Pourbaix diagrams describe equilibrium states of bulk materials, whereas the working catalyst interface exists under highly nonequilibrium conditions. Factors such as local pH gradients, adsorbate-induced surface stress, interfacial electric fields, and continuous reaction turnover collectively drive dynamic restructuring at the interface. As a result, catalyst evolution under reaction conditions is better understood as a kinetically driven and functionally relevant process rather than simple corrosion or thermodynamic instability.

Configuration changes are not limited to copper systems. Even in noble metal systems, shape-controlled Pt nanocrystals have been shown to undergo discernible structural evolution after electrochemical potential cycling, indicating that well-defined morphologies cannot be assumed to remain invariant under electrochemical conditions.^44^ Perovskite oxides also exhibit pronounced structural evolution under oxygen evolution conditions. For example, SrIrO3 undergoes oxygen-redox-driven ionic diffusion, surface amorphization, and structural reorganization during operation.^45^ Operando spectroscopic studies across hydrogen and oxygen evolution reactions further reveal dynamic reconstruction under applied potential bias.^37,46,47^ For example, operando XAS studies on hydrogen evolution catalysts have reported potential-induced rearrangement of Co–O–Ru coordination motifs, indicating continuous adjustment of local bonding environments in response to applied bias.^38^ In oxygen evolution systems, potential-dependent spectroscopic analysis further reveals the interconversion among distinct spin states in iron centers, accompanied by measurable variations in Fe–O coordination distances and redistribution of oxidation and spin states across the applied potential range.^48^ These observations demonstrate a strong coupling between electronic structure and coordination geometry, establishing that dynamic configuration changes are not limited to specific elements but represent a general response of electrocatalysts to the reaction environment.

The dynamic evolution of catalysts under reaction conditions can be broadly categorized into several representative pathways. One class involves periodic or oscillatory evolution, in which catalysts undergo reversible structural fluctuations, such as the surface breathing behavior observed in Pd during CO oxidation. A second class corresponds to irreversible restructuring, where the initial morphology is progressively altered through processes such as fragmentation, dissolution, or redeposition, as commonly observed in Cu-based electrocatalysts. A third class involves the formation of transient active phases, where dynamically generated species emerge and govern catalytic behavior. Although these pathways differ in reversibility and time scales, they collectively highlight that catalyst structures evolve continuously under nonequilibrium conditions, and that the active state is often distinct from the as-synthesized configuration. The progressive reorganization of bonding, geometry, and electronic structure links microscopic atomic events to the macroscopic evolution of catalytic form. To sum up, these collective examples evidently reveal that the as-synthesized structure does not reflect the configuration under actual reaction conditions. The atomic configurations that define catalytic behavior evolve continuously in response to illumination, thermal excitation, and electrochemical potentials. As a result, the working state of a catalyst deviates from its initial structure, and the morphology recorded prior to reaction captures only a transient snapshot rather than the configuration governing reactivity. This discrepancy challenges the traditional workflow in which well-defined nanocrystals are synthesized, characterized in the as-synthesized state, and subsequently used as structural inputs for theoretical modeling. When atomic arrangements reorganize during reaction, the theoretical descriptions computed from the initial morphology no longer represent the genuine catalytic landscape. Structure–activity interpretations based on the as-synthesized morphology may therefore misattribute catalytic behavior to local atomic configurations that do not exist in the working state. This mismatch reflects the fact that morphology provides only a well-defined starting point for reaction, rather than the atomic configurations that govern reactivity under operating conditions. Recognizing this mismatch underscores the necessity of accessing the operative morphology and developing methodologies capable of capturing how atomic configurations evolve as reactions proceed. Only by probing catalysts in their working state can morphology regain its interpretive value as a reliable descriptor of catalytic function.

Importantly, although substantial structural evolution is widely observed across photocatalytic, electrocatalytic, and thermocatalytic systems, the catalytic outcomes associated with different initial morphologies remain markedly different. For instance, shape-controlled catalysts consistently exhibit morphology-dependent activity or selectivity prior to and even during reaction, despite undergoing surface reconstruction. These observations indicate that the role of morphology cannot be dismissed simply because it evolves under operating conditions. Instead, it suggests that the initial morphology influences the pathways of structural reorganization, thereby steering the evolution toward distinct active states and catalytic behaviors. In this sense, morphology remains meaningful not as a static descriptor of the working state but as a defining factor that governs the trajectory of catalyst evolution. This interpretation does not exclude regimes in which morphology remains largely preserved under operating conditions but rather emphasizes that its validity as a structural descriptor depends on the specific reaction environment. To translate this dynamic perspective into a practical research strategy, we propose a four-step operando-informed framework for rational catalyst design (Figure 5). First, define the as-synthesized structural configuration, including facet exposure, coordination environments, and defect density, as the baseline for identifying subsequent structural evolution. Second, track operando evolution across complementary scales using time-resolved imaging and spectroscopy to monitor changes in lattice order, electronic states, and chemical composition, ensuring that structural dynamics are captured under relevant reaction conditions. Third, reconcile ensemble-averaged and local probes by integrating spatially resolved imaging with statistically robust measurements, particularly when different techniques provide seemingly inconsistent structural information. Finally, use experimentally validated working-state structures as inputs for theoretical modeling or machine-learning-assisted approaches, enabling experimentally observed dynamic structures to inform and refine theoretical descriptions beyond static models.

**Figure 5:**
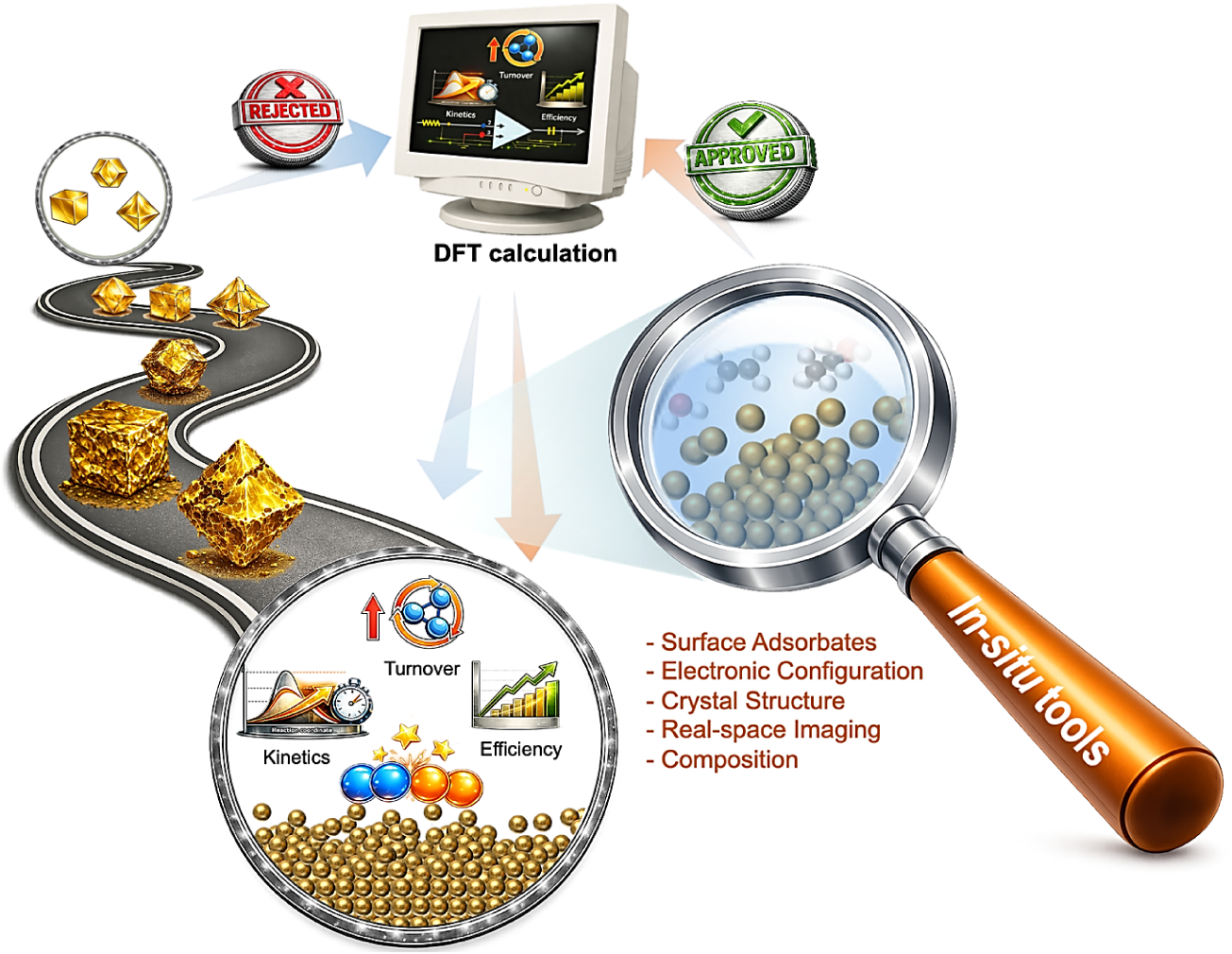
Conceptual transition from traditional catalysis workflow to an operando-informed framework. Traditional approaches rely on well-defined, static catalyst structures and theoretical models that assume structural invariance during reactions. Incorporating operando and in situ techniques enables direct observation of atomic evolution under working conditions, bridging synthesis and theory through a feedback loop grounded in experimentally verified structures.

The requirement for operando characterization to establish meaningful structure–activity relationships is inherently condition-dependent. In systems where the catalyst structure remains largely invariant under reaction conditions, as evidenced in certain cases where minimal morphological change is observed before and after reaction, as-synthesized models may serve as reasonable approximations.^49^ In contrast, operando characterization becomes essential when structural evolution is strongly coupled to the reaction environment, such as under large electrochemical overpotentials, illumination-driven charge accumulation, or high-temperature conditions that promote surface reconstruction, phase transformation, or dynamic interfacial species formation. In such cases, relying solely on ex-situ characterization can lead to misleading structure–activity correlations. This limitation may be further amplified under industrially relevant operating environments, such as in flow cells or membrane electrode assemblies, where local reaction conditions differ significantly from conventional laboratory systems.

## Make Morphology Control Meaningful: Capturing the Dynamic Evolution of Catalysts

Understanding how catalysts evolve during reactions requires methods capable of monitoring structural and electronic evolution under operating conditions rather than relying on their as-synthesized structures. Such approaches must resolve atomic motion in real time, quantify ensemble-level transformations, and track the evolution of local bonding and oxidation states under working conditions.^50^ First, real-space operando imaging provides direct visualization of structural dynamics as reactions proceed. Operando TEM and its liquid phase variants, including polymer-based electrochemical cells, enable time-resolved observation of atomic migration, lattice transformation, and transient coordination environments. These techniques have revealed processes such as the conversion of ordered oxide lattices into metallic nanograins, structural reconstructions at initially isolated sites through bond cleavage and reformation, and their reorganization into catalytically active clusters.^36,40,51^ Recent advances in polymer-based electrochemical cells have extended atomic-resolution imaging to electrified solid–liquid interfaces, where amorphous interphases fluctuate and mediate dynamic transitions.^41^ Despite their high spatial resolution, these methods remain constrained by beam-induced effects, restricted sampling volumes, and challenges in statistical representativeness. These constraints underscore the necessity of ensemble-level probes. Second, operando scattering techniques such as operando XRD and small-angle X-ray scattering spectroscopies (SAXS) provide statistically robust insight into how ordered structures evolve during catalysis. Operando XRD tracks the emergence, shift, and disappearance of diffraction features, revealing whether crystalline frameworks remain stable or undergo reconstruction under reaction conditions. These measurements enable direct correlation between crystallographic stability, surface phase formation, bulk lattice adaptation, and morphological persistence.^52,53^ SAXS complements XRD by probing mesoscale architectures, capturing dynamic changes in particle size, porosity, and structural coherence across large catalyst populations.^54^ Despite excellence at providing ensemble-level information, scattering techniques are inherently less sensitive to short-range disorder and amorphous motifs, motivating the integration of other operando spectroscopic methods that resolve local coordination and electronic environments to complete the picture of catalytic structural evolution.^54^

Third, operando XAS techniques provide element-specific access to the evolution of oxidation state, coordination environment, and electronic structure during catalytic turnover. Conventional XAS serves as a central probe of these transformations by tracking shifts in absorption edge energy and coordination number, enabling assessment of whether morphology-derived configurations persist or reorganize during reaction and whether dynamic redox equilibria sustain reactivity.^37,38,55,56^ Additionally, time-resolved or quick-scan XAS extends this capability into the millisecond domain, enabling transient oxidation-state oscillations to be resolved. The addition of temporal resolution exposes short-lived intermediates and periodic redox oscillations that drive continuous changes in local coordination environments, allowing the evolution of atomic configurations to be resolved in real time.^42,57^ Furthermore, high-energy-resolution fluorescence detection (HERFD)-XAS improves spectral definition by suppressing core-hole lifetime broadening effect, thereby significantly enhancing spectral resolution of fine spectral features that are otherwise insufficient to deconvolute in conventional measurements. This increased clarity allows overlapping electronic states and metal–ligand hybridization to be distinguished.^58,59^ HERFD-XAS thus provides unparalleled access to ligand-field distortions and the dynamic evolution of coordination symmetry.^60^ X-ray emission spectroscopy (XES) complements these absorption-based techniques by probing the occupied valence manifold. Its sensitivity to metal–ligand covalency, spin configuration, and charge redistribution enables the detection of spin-state transitions and orbital reoccupations.^48,61^ Finally, in addition to the local electronic and structural insight provided by these methods, compositional dynamics such as dissolution and redeposition may also occur during reaction. These processes can be captured by online inductively coupled plasma mass spectrometry (ICP-MS), which offers a supplementary evidence of material loss or reintegration.^62^ Concurrently, in situ vibrational spectroscopies, including IR and Raman spectroscopy, provide complementary insight into information on surface adsorbates and reaction intermediates, linking chemical evolution directly to structural dynamics.^4,35,50^ Consequently, these operando techniques collectively establish an integrated framework for monitoring catalyst morphological evolution under reaction conditions, as summarized in Figure 6. Within this framework, these methods should not be viewed as competing descriptions of the same structure, but as complementary windows onto different levels of catalyst organization. Ensemble techniques define statistically dominant phase and lattice evolution, whereas local probes reveal spatially rare yet catalytically relevant features that may be masked in averaged signals. By combining atomic-scale imaging, ensemble-averaged diffraction, electronic spectroscopy, and compositional analysis, they capture complementary aspects of structural, electronic, and chemical variations that no single method can resolve in isolation. Correlating atomic configuration, lattice order, and compositional stability in real time enables direct evaluation of whether a catalyst preserves or diverges from the state defined at synthesis. Within this integrated perspective, morphology regains its scientific relevance: not as a static structural descriptor, but as a well-defined initial condition whose transformation pathways can be quantitatively tracked and correlated with catalytic behavior. In this dynamic view, morphology control remains meaningful precisely because it defines the starting configuration from which active structures emerge and evolve under operating conditions.

**Figure 6:**
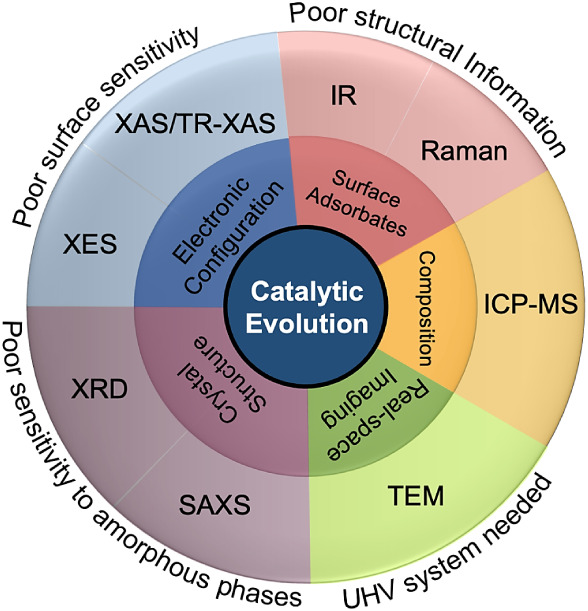
Operando characterization map for monitoring catalyst evolution under reaction conditions. The schematic summarizes complementary operando techniques used to track catalyst evolution across structural, electronic, and compositional dimensions. Techniques are positioned according to the primary information they provide, while the outer ring denotes experimental constraints that commonly accompany each class of operando technique.

## Conclusion and Outlook

Morphology control provides the structural foundation for rational catalyst design by defining well-controlled atomic configurations that guide charge distribution and reactivity. However, the atomic arrangement embodied in an initial morphology often evolves once catalysis commences, making the static structural descriptors insufficient for mechanistic interpretation. To capture how atomic configurations transform under realistic conditions, morphology design must be integrated with operando characterization capable of tracking structural evolution in real time. Through this integration, morphology control preserves its role as the starting point of catalyst design while gaining deeper significance as a dynamic framework for understanding how structure and reactivity coevolve. In this context, morphology acquires renewed value: not as a fixed geometric label, but as a well-characterized initial configuration whose evolution can be experimentally resolved and mechanistically linked to catalytic function.

The rapidly expanding landscape of operando instrumentation provides an unprecedented opportunity to build a unified, multiscale description of catalytic processes. A major challenge ahead lies in quantitatively correlating short-lived atomic rearrangements with longer-range modifications in surface architecture and lattice order. Addressing this challenge requires coordinated experimental workflows that align real-space imaging, ensemble diffraction, and element-specific spectroscopy under comparable reaction conditions, enabling more coherent interpretation of catalyst evolution.

Looking forward, the key challenge is no longer to demonstrate that catalyst morphology evolves under reaction conditions, but to transition from observation toward predictive and ultimately controllable frameworks for dynamic structural evolution. A first direction lies in moving from observation of structural evolution to predictive modeling of evolutionary trajectories. While operando techniques have revealed that catalyst structures continuously transform, the ability to predict how a given initial morphology evolves under specific reaction conditions remains limited. Future theoretical approaches should therefore move beyond static representations and aim to describe morphology as an evolving state, where the initial configuration serves as a well-defined starting point for trajectory-dependent transformations. Developing dynamic simulation frameworks, potentially integrating kinetic modeling with data-driven approaches, will be essential for enabling “design for dynamics,” in which catalyst structures are engineered not as fixed end points but as seeds of controlled evolution. A second challenge concerns the integration of multimodal operando data across different spatial and temporal scales. Although combining imaging, spectroscopy, and scattering techniques provides complementary insights, these measurements are often acquired under varying conditions and resolutions, leading to fragmented interpretations of catalyst behavior. Bridging these gaps requires the development of coordinated multimodal platforms capable of aligning structural, electronic, and chemical information across time and space. Such integration would transform isolated observations into coherent descriptions of dynamic processes, enabling a more complete understanding of catalyst evolution. Finally, evidence accumulated across different catalytic systems suggests that dynamic restructuring is not material-specific but may follow more generalizable principles. Identifying such principles requires moving beyond system-specific observations toward comparative frameworks that capture common features of structural evolution across materials. In this context, establishing cross-material data sets will be essential to distinguish structural features that persist during evolution from those that are transient. Establishing such descriptors may provide a pathway toward a more unified understanding of catalyst dynamics, potentially elevating morphology control from an empirical strategy to a predictive and mechanistically grounded design principle. From this perspective, redefining morphology as the starting point of catalytic evolution does not diminish its importance but rather expands its role in catalyst design. The central challenge ahead lies in bridging the gap between tracking structural dynamics and achieving predictive control over evolutionary pathways. When catalyst evolution can be anticipated and guided, morphology will no longer be viewed as a static structural attribute but as a dynamic parameter that can be deliberately engineered to govern catalytic function under working conditions.
